# Application of polyvinylpyrrolidone-stabilized silver nanoclusters as fluorescent probes for trace iodide in seaweed

**DOI:** 10.3389/fnut.2026.1804626

**Published:** 2026-04-30

**Authors:** Xueling Cao, Fei Li, Tingting Zheng, Guohui Li, Danfeng He

**Affiliations:** 1 College of Science, Qiongtai Normal University, Haikou, Hainan, China; 2 College of Food and Biological Sciences, Jilin University of Chemical Technology, Jilin, China

**Keywords:** fluorescent probes, iodide detection, mechanism analysis, seaweed, silver nanoclusters

## Abstract

**Introduction:**

Iodide is an essential trace nutrient in foods like seaweed, seafood, and iodized salt, with balanced intake critical for thyroid function and metabolic health. Accurate trace detection is key for nutrition evaluation and iodide deficiency prevention. Existing methods often face challenges in complex food matrices, so this study aimed to construct a polyvinylpyrrolidone (PVP)-stabilized silver nanocluster (AgNCs@PVP) fluorescent probe for trace iodide detection in seaweed.

**Methods:**

AgNCs@PVP probes were synthesized via chemical reduction with PVP as a stabilizer, characterized by UV–Vis, TEM, and fluorescence spectroscopy. The iodide detection system was optimized for pH, reaction time, and probe concentration. Seaweed samples were pretreated by crushing, soaking, microwave-assisted extraction, protein precipitation, centrifugation, and pH adjustment. Sample solutions were mixed with AgNCs@PVP, and fluorescence intensity was measured at 340 nm excitation. Linear range, detection limit, selectivity, recovery, and repeatability were evaluated.

**Results:**

Iodide significantly quenched AgNCs@PVP fluorescence, showing a good linear response (0.29 μM–280 μM) with a detection limit of 56 nM. Common interfering substances in seaweed had no obvious impact on detection. The system exhibited good recovery (92.3–105.6%) and repeatability (RSD < 3.0%) in seaweed sample analysis. Synthesized AgNCs@PVP were spherical, uniformly dispersed (average size ~3.3 nm), and had stable fluorescence properties.

**Discussion:**

The AgNCs@PVP probe integrates PVP’s stabilizing effect and AgNCs’ excellent fluorescence performance, achieving sensitive and selective iodide detection. Its low detection limit and wide linear range address the needs of trace analysis in complex seaweed matrices, overcoming the drawbacks of traditional methods (cumbersome operation, poor selectivity). This study provides a rapid, reliable method for food iodide detection, supporting rational iodide intake and food nutritional safety.

## 1 Introduction

Iodine is an essential trace nutrient for the human body, playing an irreplaceable role in the synthesis of thyroid hormones, regulation of metabolic balance, and normal nervous system development (1). It is widely present in food sources such as seaweed, seafood, iodized salt, and dairy products, as well as in environmental water and biological samples. However, both insufficient and excessive iodide intake can pose health risks: iodide deficiency may lead to goiter, intellectual disabilities, and abnormal growth and development, while excessive iodide intake can induce thyroid inflammation, hyperthyroidism, and other thyroid diseases (2). Therefore, accurately detecting trace iodide ions in food and environmental samples is crucial for food nutrition assessment, clinical nutrition monitoring, and the prevention and control of iodide-related diseases, making it a topic of broad interest in academia and public health.

To meet this critical detection need, various methods for the quantitative analysis of iodide ions have been developed, including inductively coupled plasma mass spectrometry (ICP-MS) (3), gas chromatography (GC) (4), electrochemical methods (5, 6), and spectrophotometry (7). Among these techniques, ICP-MS offers ultrahigh sensitivity and a wide detection range, but it relies on expensive instruments and specialized operation, which limits its application in rapid on-site detection. GC methods typically require iodide derivatization, a process that is time-consuming and cumbersome. Electrochemical methods have the advantages of fast response and low cost, but the presence of coexisting substances in complex matrices can easily affect their detection stability, resulting in poor reproducibility. Spectrophotometry is widely used for routine detection due to its simple operation and low cost, but its low sensitivity and poor selectivity make it difficult to meet the detection requirements for trace iodide ions in complex samples. Therefore, developing a detection technology that is highly sensitive, selective, easy to operate, and low-cost remains an urgent task in iodide ion analysis.

Owing to its outstanding advantages such as high sensitivity, rapid response, simple operation, and great potential for visualization, fluorescence detection shows broad application prospects in the field of trace substance detection (8). Traditional, organic, small-molecule fluorescent probes have issues such as poor photostability, susceptibility to environmental pH, and poor biocompatibility (9). Although quantum dot fluorescent probes have good photostability and high fluorescence quantum yield, they have potential biological toxicity and complex preparation processes, which limit their practical applications (10). As a new type of fluorescent nanomaterial, metal nanoclusters have become a research hotspot in the field of fluorescent probes because of their unique advantages, including ultrasmall size (usually less than 2 nm), good photostability, low biological toxicity, high fluorescence quantum yield, and ease of functional modification (11). Among them, silver nanoclusters (AgNCs) have attracted widespread attention from researchers because of their low raw material cost, diverse preparation methods, and tunable fluorescence properties.

The fluorescence performance and stability of AgNCs are highly dependent on the choice of stabilizers, which commonly include proteins, nucleic acids, thiol compounds, and polymers (12–15). As a water-soluble polymer, polyvinylpyrrolidone (PVP) has good biocompatibility, chemical stability, and dispersibility. The pyrrolidone groups in its molecular chains can coordinate with Ag^+^, effectively inhibiting AgNC aggregation while providing a stable microenvironment that regulates their fluorescence properties (16, 17). Compared with biomacromolecular stabilizers such as proteins and nucleic acids, PVP is more cost-effective, easily obtainable, and simpler to prepare; compared with thiol stabilizers, PVP-modified AgNCs (AgNCs@PVP) exhibit better water solubility and storage stability, and their fluorescence is less likely to be quenched because of the oxidation of thiol groups (18, 19).

In this study, PVP was used as a protective agent to prepare AgNCs@PVP fluorescent probes via a simple chemical reduction method. The silver nanoclusters were thoroughly characterized. Given the advantage of the quenching effect of iodide on the fluorescence signal of AgNCs@PVP, a highly sensitive fluorescent detection system for iodide was established, and the detection conditions were optimized to improve the selectivity and accuracy of the method. The established detection method was then applied to the quantitative analysis of iodide in sausage samples to verify its practical performance. In addition, the fluorescence quenching mechanism between AgNCs@PVP and iodide was further investigated, providing theoretical support for the design and performance optimization of such fluorescent probes. Overall, this work seeks to develop a novel, efficient, and practical fluorescent detection technology for trace iodide, offering new strategies for food nutrition evaluation and environmental monitoring.

## 2 Materials and methods

### 2.1 Reagents and instruments

PVP K15 was obtained from Sigma–Aldrich. Silver nitrate and formaldehyde were procured from Tianjin Yongda Chemical Reagent Co., Ltd. Potassium dichromate was purchased from Shanghai Zhanyun Chemical Co., Ltd. All reagents were analytically pure and did not require further processing.

The following types of equipment were used: RF-5301PC fluorescence spectrophotometer (Shimadzu, Japan) and HT7800 transmission electron microscope (Hitachi, Japan).

### 2.2 Preparation of AgNCs@PVP

In brief, 1 mL of 36 mg/mL silver nitrate, 1 mL of 12 mg/mL PVP, and 10 mL formaldehyde aqueous solution were accurately added into a polytetrafluoroethylene reactor, and the mixture was stirred for 1 min. The system pH was adjusted to 1. Afterward, the mixture was placed in an oven for reaction at 180 °C for 18 h and then in a dialysis bag with a molecular weight of 20,000 Da for 32 h. Finally, the purified solution was freeze-dried to obtain a light yellow solid powder (16).

### 2.3 Determination of interfering species

The following interfering substances were separately added at 1 mg/mL 100 μL into 0.5 mg/mL AgNCs@PVP: Na^+^、K^+^、Ca^2+^、Mg^2+^、Fe^2+^、Fe^3+^、Zn^2+^、Mn^2+^、Cu^2+^、Al^3+^、Cl^−^、SO₄^2−^、NO₃^−^、NO₂^−^、PO₄^3−^ and Br^−^. Measure the fluorescence emission intensity of AgNCs@PVP using a fluorescence spectrophotometer with an excitation wavelength set at 340 nm.

### 2.4 Actual seaweed sample treatment

The extraction and purification processes of iodide ions were modified from the literature (20). First, the dried seaweed samples were crushed, ground into fine powder, and passed through an 80-mesh sieve to ensure uniformity. Then, 1.000 g of the sieved seaweed powder was accurately weighed and placed in a 50 mL centrifuge tube, followed by the addition of 20 mL ultrapure water for soaking and swelling for 30 min. After adjusting the pH of the mixture to 2.5 with 1 mol/L hydrochloric acid, microwave-assisted extraction was performed at 300 W and 70 °C for 5 min. Subsequently, 1.0 mL of 10% potassium ferrocyanide solution and 1.0 mL of 20% zinc acetate solution were added sequentially to precipitate proteins and polysaccharides, and the mixture was centrifuged at 8000 r/min for 15 min at 4 °C. The obtained supernatant was filtered through qualitative filter paper, and the filtrate was transferred to a 50 mL volumetric flask and made up to volume with ultrapure water. A 10 mL aliquot of the diluted filtrate was taken and adjusted to pH 7.0 with 1 mol/L sodium hydroxide solution to obtain the sample solution for detection. Finally, the sample solution was mixed with AgNCs@PVP fluorescent probe and shaken thoroughly. After standing at room temperature for 5 min, the fluorescence emission intensity of AgNCs@PVP was measured at an excitation wavelength of 340 nm.

## 3 Results and discussion

### 3.1 Characterization of AgNCs@PVP

**Figure 1:**
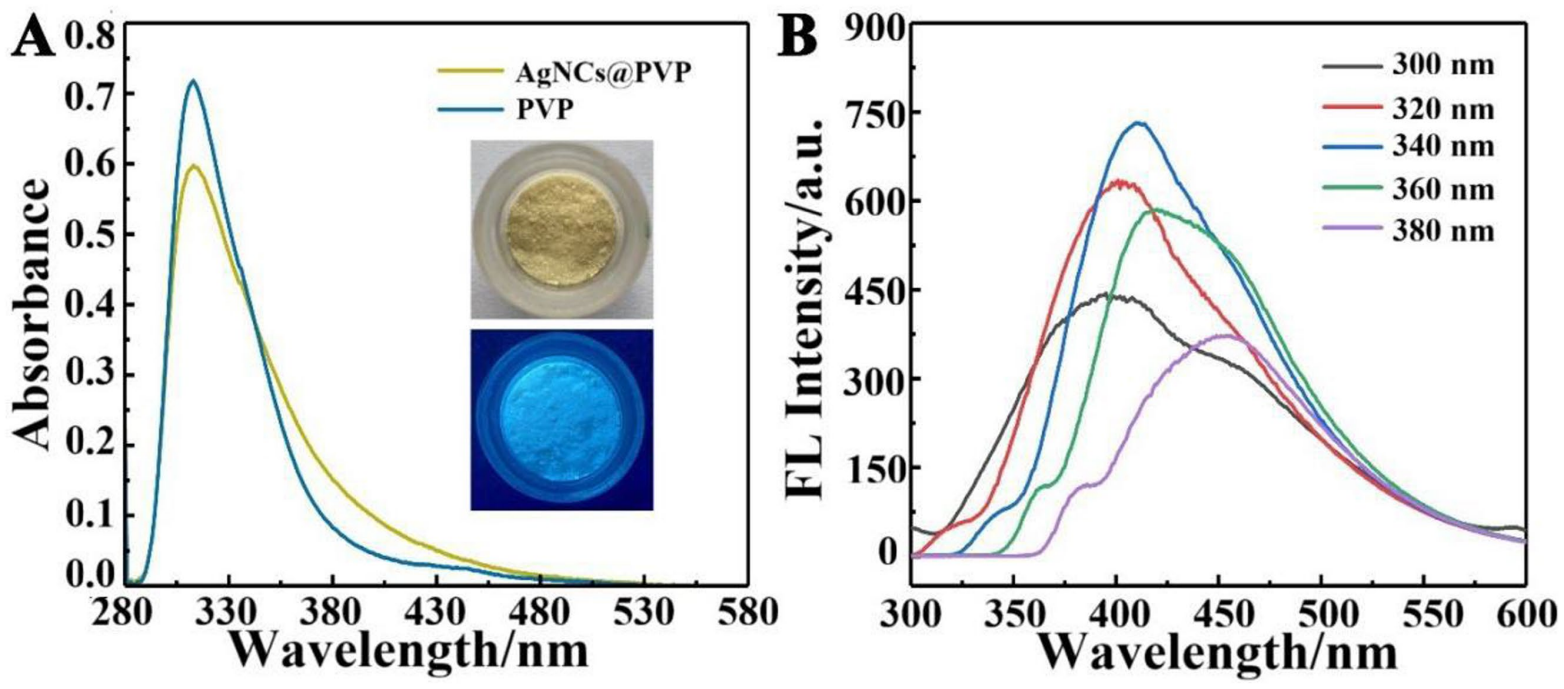
**(A)** UV–Vis absorption spectra of AgNCs@PVP and PVP; **(B)** Emission spectra of AgNCs@PVP at different excitation wavelengths.

**Figure 2:**
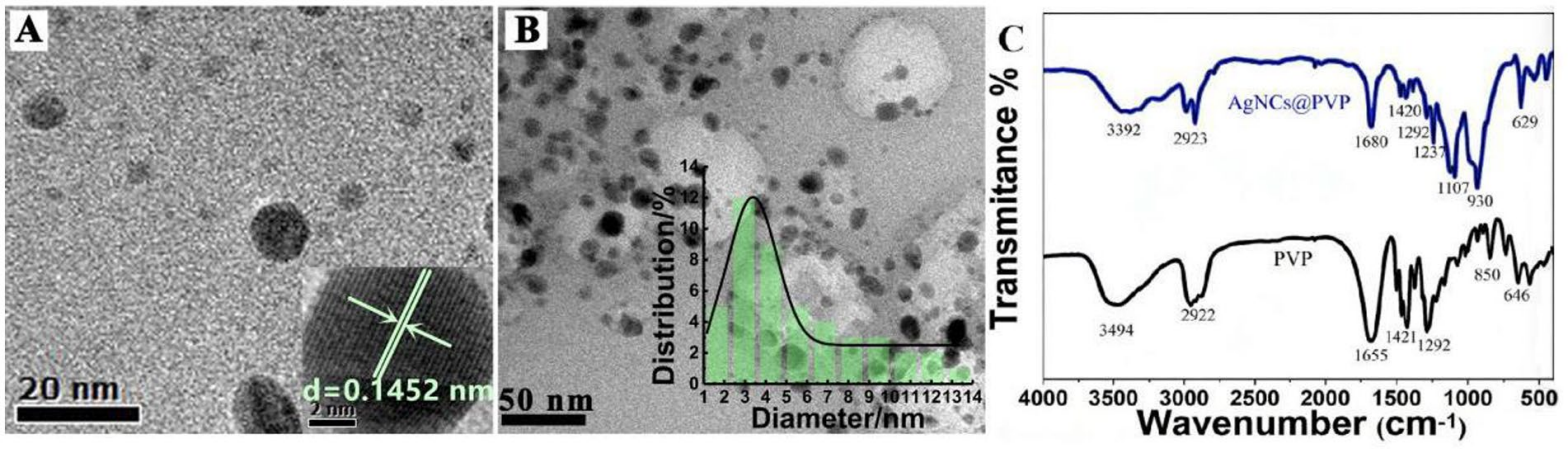
**(A)** HRTEM (20 nm) of AgNCs@PVP; **(B)** HRTEM diagram (50 nm) of AgNCs@PVP; **(C)** FT-IR spectrum of AgNCs@PVP and PVP.

Figure 1A shows the absorption spectra of AgNCs@PVP and its ligand PVP. The maximum absorption peak is located at 320 nm, corresponding to AgNCs@PVP. No plasmon resonance absorption peak is observed, indicating that small AgNCs were synthesized (21). Figure 1B shows the emission spectra of AgNCs@PVP under different excitation wavelengths, with the emission peak around 420 nm. The fluorescence intensity of AgNCs@PVP is the highest when the excitation wavelength is 340 nm. The morphology of AgNCs@PVP was analyzed using transmission electron microscopy to further confirm its formation. Figure 2A shows the HRTEM image of AgNCs@PVP, with a calculated lattice spacing of 0.1452 nm. This lattice spacing is close to the (202) lattice spacing of AgNCs at 0.1457 nm and should be attributed to the (202) plane of AgNCs. As shown in Figure 2B, the prepared AgNCs@PVP are spherical and distributed relatively uniformly. According to the particle size distribution of AgNCs@PVP, most particles are between 1.5 and 5.5 nm, with an average particle size of approximately 3.3 nm. To confirm the chemical interaction between AgNCs and PVP in composites, Fourier transform infrared spectroscopy (FT-IR) testing was performed. As shown in Figure 2C, the O-H/N-H expansion peak of PVP at 3494 cm^−1^ moves to 3,392 cm^−1^ in the AgNCs@PVP, indicating hydrogen bonding or coordination between AgNCs and the polar groups of PVP. At the same time, the C=O telescopic peak blue of PVP at 1655 cm^−1^ was moved to 1,680 cm^−1^, which verified the coordination between PVP carbonyl groups and Ag atoms on the surface of AgNCs. The slight shift of the C-H (approximately 2,922 cm^−1^) and C-N (1,292 cm^−1^) peaks indicates that the main backbone of PVP remains intact, while its functional groups stabilize AgNCs. These results provide direct spectroscopic evidence for the successful preparation of AgNCs@PVP and the interfacial chemical interactions between its two components. The UV–Vis absorption spectrum shows no obvious surface plasmon resonance (SPR) peak at 400–450 nm, indicating that very few large silver nanoparticles were formed in the system. Combined with the transmission electron microscopy (TEM) results, which directly show ultra-small and uniformly dispersed particles, it confirms that well-distributed silver nanoclusters (AgNCs) have been successfully synthesized.

**Figure 3:**
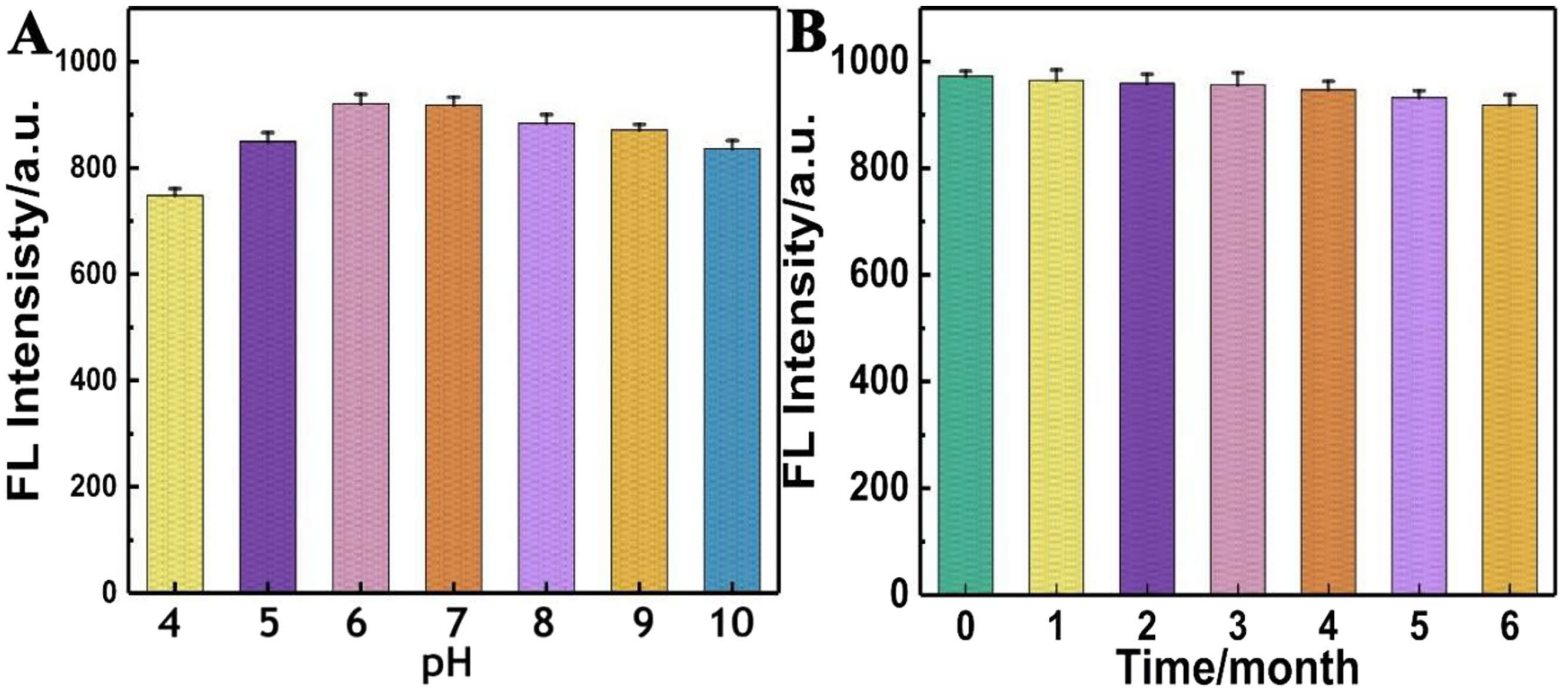
**(A)** Effect of different pH values on the fluorescence intensity of AgNCs@PVP; **(B)** Effect of different storage times on the fluorescence intensity of AgNCs@PVP.

Based on previous work (16), the optimized experimental reaction conditions were directly used for the preparation of AgNCs@PVP in this experiment. After preparing several batches, the stability of AgNCs@PVP at different pH values was examined. As shown in Figure 3A, within the pH range of 4–10, there was no significant change in the fluorescence intensity of AgNCs@PVP, indicating that the environmental pH has little effect on their stability. Figure 3B shows the effect of different storage times of the prepared AgNCs@PVP on their fluorescence intensity. It can be seen from the figure that after 6 months of storage, the fluorescence intensity of AgNCs@PVP did not decrease, indicating that AgNCs@PVP have good stability and are suitable for future practical applications.

### 3.2 Establishment of the iodide detection method by AgNCs@PVP

**Figure 4:**
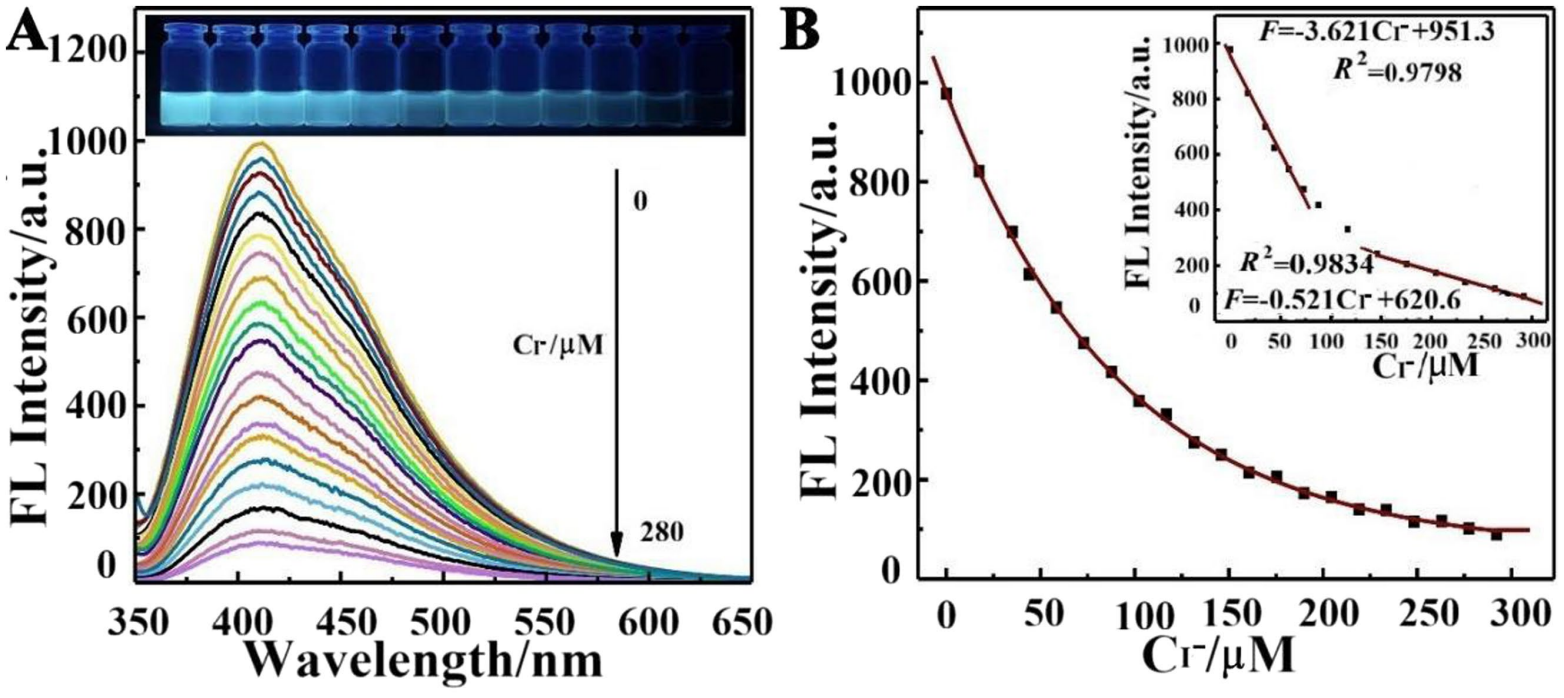
**(A)** Fluorescence emission spectrum of AgNCs@PVP with different concentrations of iodide; **(B)** Relationship *F* of AgNCs@PVP with different concentrations of iodide (inset: the standard curve).

As shown in Figure 4A, AgNCs@PVP exhibits a significant fluorescence emission peak at 420 nm, and blue fluorescence can be observed under a 365 nm UV lamp. When iodide solutions of different concentrations are added to the prepared AgNCs@PVP, the fluorescence intensity of AgNCs@PVP gradually decreases with the increase in iodide concentration. When the concentration reaches 280 μM, the fluorescence is almost completely quenched, indicating a strong interaction between iodide and AgNCs@PVP. Figure 4B shows that within the concentration range of 0.29–280 μM, a good linear relationship exists between the fluorescence intensity of AgNCs@PVP and iodide concentration. In the range of 0.29–86 μM, the linear equation is *F* = −3.621[iodide] + 951.3, with a linear correlation coefficient *R^2^* = 0.9798. In the range of 86–280 μM, the linear equation is *F* = −0.521 [iodide] + 620.6, with a linear correlation coefficient *R^2^* = 0.9834. The detection range of AgNCs@PVP for iodide is 0.29–280 μM, with a detection limit of 56 nM.

### 3.3 Interference test

**Figure 5:**
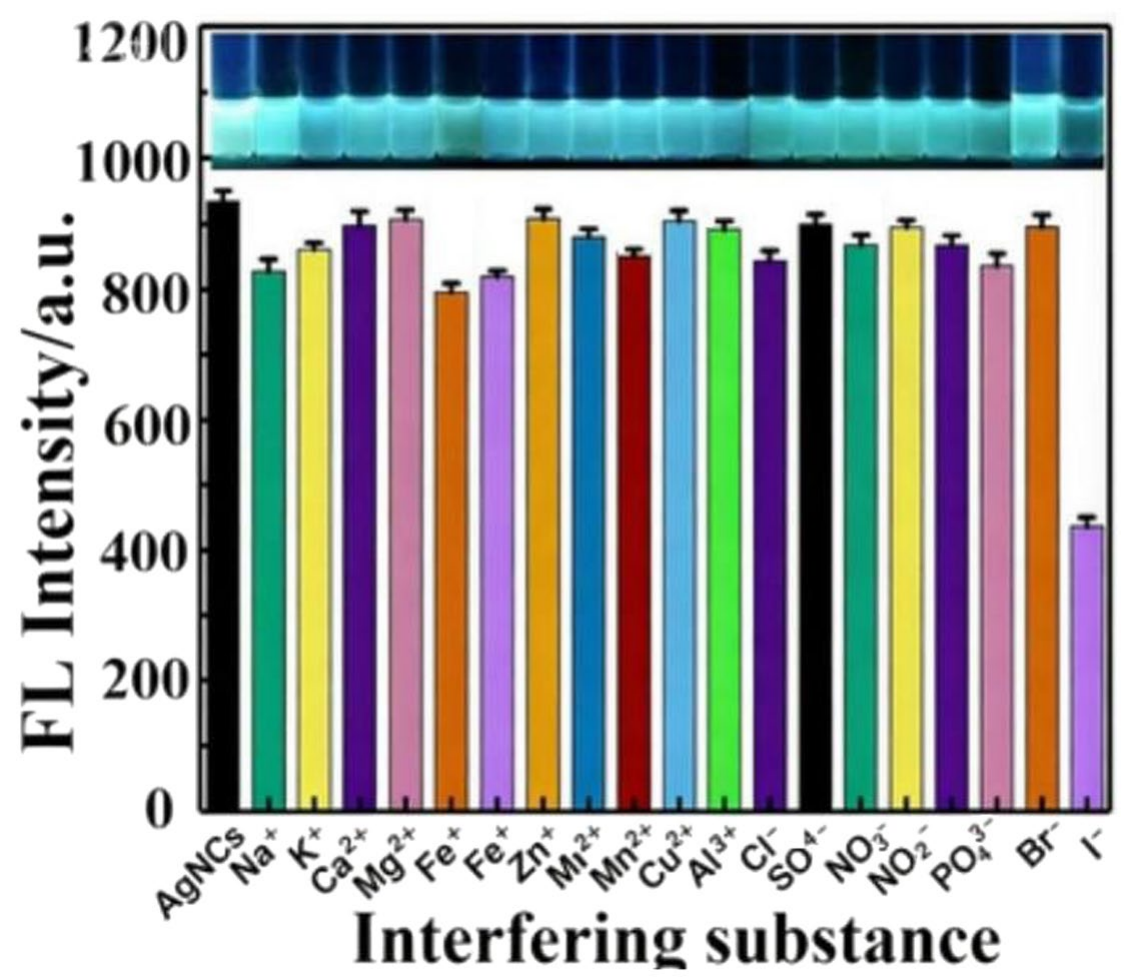
The fluorescence intensity changes of AgNCs@PVP mixed with different interfering species.

The established fluorescence detection method was applied to actual samples to further investigate the potential effects of interfering substances Na^+^, K^+^, Ca^2+^, Mg^2+^, Fe^2+^, Fe^3+^, Zn^2+^, Mn^2+^, Cu^2+^, Al^3+^, Cl^−^, SO₄^2−^, NO₃^−^, NO₂^−^, PO₄^3−^, and Br^−^ on the fluorescence emission intensity of AgNCs@PVP (22). The experimental results showed that in the presence of interfering ions at the same concentration (0.05 mg/mL), only iodide has a significant impact on the fluorescence emission spectrum of AgNCs@PVP (Figure 5). This finding indicates that AgNCs@PVP has high selectivity for iodide, which is favorable for its application in actual samples.

### 3.4 Mechanism of iodide detection by AgNCs@PVP

**Figure 6:**
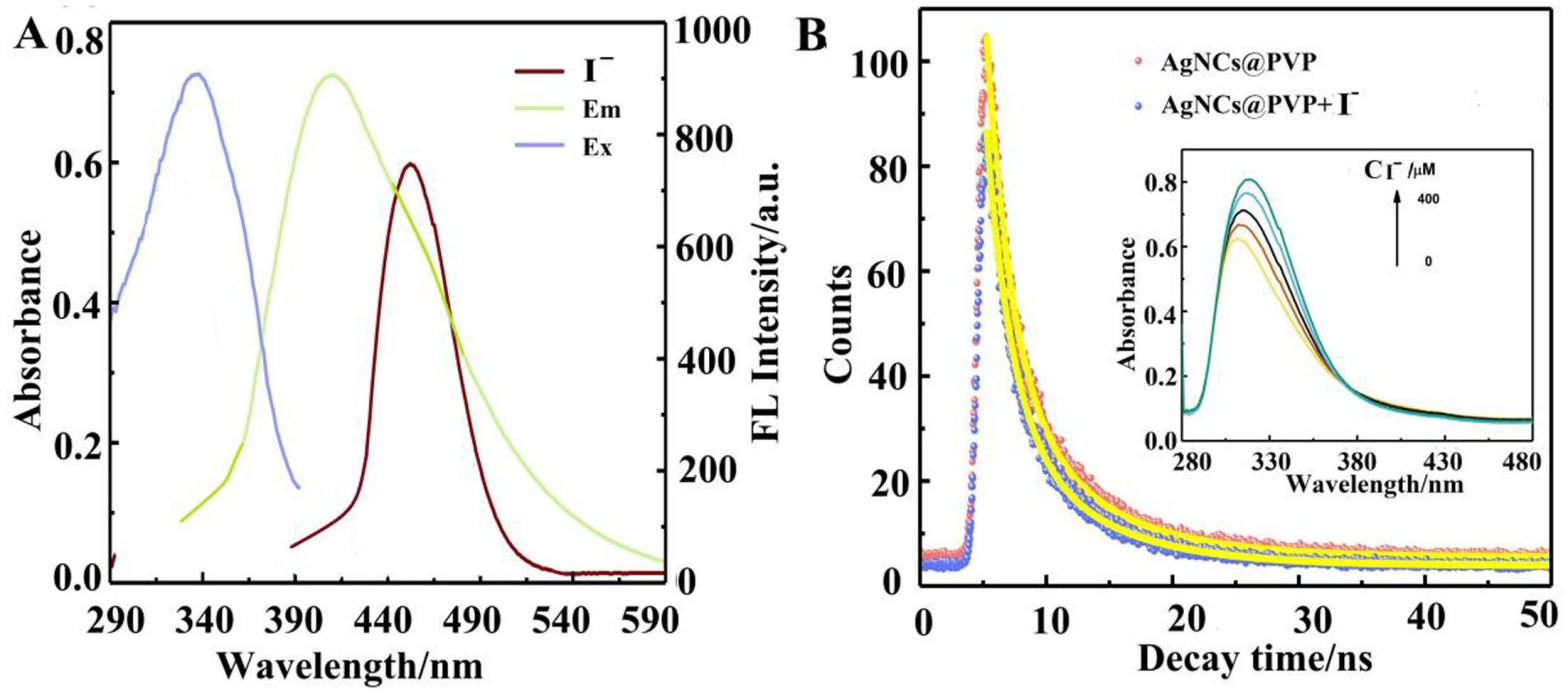
**(A)** UV–Vis absorption spectra of iodide, AgNCs@PVP, and AgNCs@PVP-iodide system, excitation and emission spectra of AgNCs@PVP; **(B)** Fluorescence lifetime curves of AgNCs@PVP and AgNCs@PVP-iodide (inset shows the effect of adding different concentrations of iodide on the UV absorption spectra of AgNCs@PVP).

The mechanism by which iodide causes the fluorescence quenching of AgNCs@PVP was further investigated. The mechanisms leading to fluorescence probe quenching are mainly divided into three categories: inner filter effect (IFE), photoinduced electron transfer, and fluorescence resonance energy transfer (FRET). As an electron donor, PVP induces emission quenching through donor–acceptor interactions (neutral nitrogen atoms on the surface of AgNCs@PVP acting as the donor). The emission spectrum of AgNCs@PVP hardly overlaps with the absorption spectrum of iodide in aqueous solution, ruling out the possibility of FRET (Figure 6A). In addition, the UV–visible absorption spectrum of iodide significantly overlaps with the excitation spectrum of AgNCs@PVP, indicating that the fluorescence quenching is caused by the IFE.

First, the spectral overlap between AgNCs@PVP and I^−^ was analyzed to determine the possibility of FRET and IFE. As shown in Figure 6A, the emission spectrum of AgNCs@PVP (400–500 nm) hardly overlaps with the absorption spectrum of I^−^ in aqueous solution (200–350 nm), with the spectral overlap integral close to 0, thus excluding the possibility of FRET occurring between AgNCs@PVP and I^−^ (23). In addition, the UV–Vis absorption spectrum of I^−^ (250–350 nm) significantly overlaps with the excitation spectrum of AgNCs@PVP (300–400 nm), and the overlap range covers the optimal excitation wavelength of AgNCs@PVP (340 nm), indicating that the fluorescence quenching may be caused by IFE (24). IFE is a common fluorescence quenching mechanism, resulting from the analyte absorbing the excitation or emission light of the fluorescent probe, leading to a decrease in effective excitation light or a reduction in detected emission.

To further quantify the contribution of IFE to the total fluorescence quenching, the classic fluorescence intensity correction formula for IFE was used for calculation (24):


$$F_{\mathrm{cor}}=F_{\mathrm{obs}}\times 10^{(A_{\mathrm{ex}}+A_{\mathrm{em}})/2}.$$

Here, F_cor_ and F_obs_ represent the corrected fluorescence intensity and observed fluorescence intensity of AgNCs@PVP after the addition of I^−^, respectively; A_ex_ and A_em_ are the absorbances of I^−^ at the excitation wavelength (340 nm) and emission wavelength (420 nm) of AgNCs@PVP, respectively. Quantitative calculation results show that at the maximum I^−^ concentration (280 μM), IFE accounts for approximately 37.7% of the total fluorescence quenching, while the remaining 62.3% of the quenching is attributed to other quenching mechanisms (25), indicating that IFE is one of the mechanisms of fluorescence quenching but not the major one.

The effect of adding 150 μM iodide on the fluorescence lifetime of AgNCs@PVP was examined to explore the mechanism underlying the iodide-induced fluorescence quenching of AgNCs@PVP, as shown in Figure 6B. The prepared AgNCs@PVP shows fluorescence lifetimes (τ1/τ2) of 2.0134 ns (34.15%)/7.0827 ns (65.85%), with an average PL lifetime of 5.35 ns. After the addition of 400 μM iodide, the average fluorescence lifetime of the AgNCs@PVP–iodide system is 5.17 ns, showing minimal changes. This finding suggests that the process is primarily static quenching. As shown in the inset of Figure 6B, the maximum absorbance of AgNCs@PVP increases after the addition of different concentrations of iodide. This result indicates that the ground-state molecules of AgNCs@PVP may form complexes with iodide, further confirming that the interaction between AgNCs@PVP and iodide is a static quenching process. In summary, the quenching of AgNCs@PVP fluorescence by iodide is caused jointly by IFE and static quenching.

### 3.5 Determination of iodide in actual samples

The established method was applied to the quantitative detection of I^−^ in real seaweed samples. A three-level spike recovery experiment showed a recovery rate of 92.3–105.6% (RSD < 3.0%). The detection results were consistent with the ICP-MS certified reference method, verifying that the method has good accuracy and reliability. The AgNCs@PVP fluorescent probe has advantages such as simple synthesis, low cost, high sensitivity, good selectivity, and stable performance. The detection method is simple to operate, fast, and does not require expensive instruments, overcoming the shortcomings of traditional iodide detection methods, which are cumbersome, have poor selectivity, and high instrument costs. This study provides a novel and efficient method for trace detection of I^−^ in seaweed and other food samples, and also provides a reference for the design and development of metal nanocluster-based fluorescent probes for the detection of trace nutritional elements (see Table 1).

**Table 1:** Application of the fluorescence method and ICP-MS for the determination of iodide in seaweed samples.

Sample no.	Spiked level (μM)	Detected by FL/μM	Detected by ICP-MS/μM	Consistent/%	Recovery/%
1–1	1.0	1.020	0.098	96.08	102.1
1–2	1.0	0.951	0.092	96.84	94.5
1–3	1.0	1.013	0.097	96.04	101.3
2–1	10.0	10.12	0.987	97.53	101.2
2–2	10.0	9.232	0.905	97.84	92.3
2–3	10.0	10.35	1.012	97.78	103.5
3–1	100.0	105.6	10.23	96.85	105.6
3–2	100.0	95.80	9.32	97.28	95.8
3–3	100.0	102.3	9.98	97.55	102.3

## 4 Conclusion

AgNCs@PVP with good luminescent properties was synthesized using PVP as a ligand, and a method was developed to detect iodide using AgNCs@PVP as a fluorescent probe. The detection range of this method is 0.29–280 μM with a detection limit of 56 nM. It was then applied to determine the iodide content in actual samples. The synthesis of AgNCs@PVP involves simple operational steps and mild preparation conditions. Meanwhile, the detection method using AgNCs@PVP exhibits a low detection limit and high sensitivity, providing a feasible approach for detecting iodide in seaweed.

## Data Availability

The original contributions presented in the study are included in the article/supplementary material, further inquiries can be directed to the corresponding authors.
